# Infant Formula Feeding at Birth Is Common and Inversely Associated with Subsequent Breastfeeding Behavior in Vietnam^1^,^2^,^3^

**DOI:** 10.3945/jn.116.235077

**Published:** 2016-09-07

**Authors:** Tuan T Nguyen, Mellissa Withers, Nemat Hajeebhoy, Edward A Frongillo

**Affiliations:** 4 Alive & Thrive, Hanoi, Vietnam;; 5 Keck School of Medicine, University of Southern California, Los Angeles, CA; and; 6 Arnold School of Public Health, University of South Carolina, Columbia, SC

**Keywords:** breastfeeding cessation, infant formula, infants and young child feeding, prelacteal feeding, Vietnam

## Abstract

**Background:** The association between infant formula feeding at birth and subsequent feeding patterns in a low- or middle-income context is not clear.

**Objective:** We examined the association of infant formula feeding during the first 3 d after birth with subsequent infant formula feeding and early breastfeeding cessation in Vietnam.

**Methods:** In a cross-sectional survey, we interviewed 10,681 mothers with children aged 0−23 mo (mean age: 8.2 mo; 52% boys) about their feeding practices during the first 3 d after birth and on the previous day. We used stratified analysis, multiple logistic regression, propensity score-matching analysis, and structural equation modeling to minimize the limitation of the cross-sectional design and to ensure the consistency of the findings.

**Results:** Infant formula feeding during the first 3 d after birth (50%) was associated with a higher prevalence of subsequent infant formula feeding [stratified analysis: 7−28% higher (nonoverlapping 95% CIs for most comparisons); propensity score-matching analysis: 13% higher (*P* < 0.001); multiple logistic regression: OR: 1.47 (95% CI: 1.30, 1.67)]. This practice was also associated with a higher prevalence of early breastfeeding cessation (e.g., <24 mo) [propensity score-matching analysis: 2% (*P* = 0.08); OR: 1.33 (95% CI: 1.12, 1.59)]. Structural equation modeling showed that infant formula feeding during the first 3 d after birth was associated with a higher prevalence of subsequent infant formula feeding (β: 0.244; *P <* 0.001), which in turn was linked to early breastfeeding cessation (β: 0.285; *P <* 0.001).

**Conclusions:** Infant formula feeding during the first 3 d after birth was associated with increased subsequent infant formula feeding and the early cessation of breastfeeding, which underscores the need to make early, exclusive breastfeeding normative and to create environments that support it.

## Introduction

Suboptimal breastfeeding and complementary feeding practices are associated with a high prevalence of malnutrition and child mortality around the world, especially in low- and middle-income countries (LMICs) (1–3). Recent global estimations have shown that suboptimal breastfeeding practices are associated with ∼800,000 deaths annually in children aged <5 y (2, 3) and economic losses of $302 billion (4). Despite many efforts to promote breastfeeding, breastfeeding practices have remained suboptimal worldwide: the prevalence of early initiation of breastfeeding among children aged <2 y is 44%, exclusive breastfeeding among children <6 mo is 38%, and continued breastfeeding at 2 y is 49% (1–3). Breastfeeding prevalence is even lower in the most populous regions of East Asia and the Pacific (including China). In 2011, the prevalence of early initiation of breastfeeding in Vietnam was 40%, exclusive breastfeeding <6 mo was 17%, and continued breastfeeding at 2 y was 19% (1, 5).

Regardless of the guidelines and efforts to protect, promote, and support breastfeeding in health facilities (6), feeding infant formula to newborns in hospitals is still common in both developed and developing countries (7–13). For example, data from the past 10 y have shown that the prevalence of this practice was 82% in Hong Kong, 67% in Canada, 55% in Vietnam, ∼50% in the United States, and 33% in Australia (7–13); mothers’ intention to feed infant formula was associated with early, exclusive, and continued breastfeeding (8, 11). The prevalence would even be higher in LMICs if mothers used infant formula to replace other prelacteal foods (8, 14). In the past 2 decades, the promotion and sales of infant formula around the world have skyrocketed, with the largest growth in LMICs (4, 15–17). Although the International Code of Marketing of Breastmilk Substitutes (18) was released ∼35 y ago, challenges with implementation, monitoring, and compliance persist worldwide, especially in LMICs (4, 15, 19).

The association between the early introduction of infant formula with the prevalence of exclusive and prolonged breastfeeding and the subsequent use of infant formula is unclear. Observational studies from high-income countries show that in-hospital feeding of infant formula is associated with formula feeding at the time of discharge and early breastfeeding cessation (7, 11, 13, 16, 20–23), whereas a randomized controlled trial (RCT) in North America showed the opposite (24). Information bias of an observational study is a key issue; the bias can occur when a mother has poor recall, the desire to give a socially acceptable response, or differential recall related to subsequent feeding practices (25). In addition, infant formula feeding at birth might be a marker of a weak commitment to breastfeeding rather than a cause of subsequent breastfeeding practices (26). Although an RCT can theoretically overcome this methodologic issue, it is not easy or ethical to randomly assign newborns to receive infant formula unless there are specific medical conditions involved (24, 27). In addition, the generalizability of an RCT might be limited if its sample is small or not representative (24).

We analyzed data from a large-scale cross-sectional survey in Vietnam to examine the association of infant formula feeding during the first 3 d after birth with subsequent feeding practices in children aged <24 mo. We hypothesized that infant formula feeding at birth would be associated with an increased prevalence of infant formula feeding and early breastfeeding cessation during the first 2 y of life. We used stratified analysis, multiple logistic regression, propensity score-matching analysis, and structural equation modeling to minimize the limitation of the cross-sectional design and to ensure the consistency of the findings.

## Methods

### 

#### Participants.

Between July and August 2011, we interviewed 11,021 mothers of children aged <24 mo who had participated in a household survey in 11 of 63 provinces or 4 of 6 ecological regions in Vietnam. This was a baseline survey of Alive & Thrive, an initiative to save lives, prevent illness, and ensure healthy growth and development through improved breastfeeding and complementary feeding practices (28). Detailed information about the determination of sample size, sampling procedure, data collection procedures, and data quality control has been described elsewhere (29). Briefly, mothers were recruited using a 3-stage cluster sampling technique that selected *1*) intervention and comparison districts, *2*) primary sampling units (equivalent to an average-sized village) based on the population-proportionate-to-size method, and *3*) mother-child dyads with the use of birth registry systematic sampling (29). During recruitment, mothers who were not in town (<5%) were replaced with alternates from a pre-identified list. The response rate was 98% among mothers who were in town. For this analysis, we excluded children born at home (*n* = 274) and who had never been breastfed (*n* = 52). We also excluded children for whom there was no information about their mother’s intention of feeding infant formula at birth (*n* = 11) and whose measured weight (*n* = 3) was not obtained. The total sample size for the analysis was 10,681.

#### Outcome variables.

Two outcome variables were subsequent feeding of infant formula—having fed the child infant formula the day before the survey—and early breastfeeding cessation (e.g., <24 mo)—not having fed breast milk to the child the day before the survey. This information was collected with the use of the standard questionnaire for indicators of infant and young child feeding recommended by the WHO (30).

#### Exposure variables.

Infant formula feeding during the first 3 d after birth was defined based on mothers’ recall. Mothers were asked to indicate from a question read aloud whether they had given their children any plain water, sugar or glucose, honey, infant formula, or anything else in addition to breastfeeding. Children who had been given infant formula were categorized as having received it within the first 3 d after birth. A similar question about prelacteal feeding has been used in various nationally representative surveys worldwide, including the Demographic and Health Surveys and UNICEF’s Multiple Indicator Cluster Surveys (5, 8, 31–33). In Vietnam, mothers and family members know about the types of foods given to their newborns, even when the foods are given by a health worker.

#### Covariates.

Child age and sex were obtained from face-to-face interviews with mothers. Child weight-for-age *z* scores were estimated based on measured weight, child age, and sex with the use of WHO growth standards (34). Intention to feed infant formula at birth was indicated for mothers who responded “yes” to the following question: “Did you or your family member bring any infant formula to the health facility when you went to give birth to (NAME)?” No professional breastfeeding support within the first 3 d after birth was indicated when mothers did not receive breastfeeding support by a health worker (e.g., doctor, nurse, or midwife) within the first 3 d after birth. Whether an infant formula promoted on television had been seen within the last 30 d was indicated when mothers answered “yes” to the following question: “During the last 30 d, have you seen any advertisements about infant formula on television?”

Breastfeeding misconceptions were based on 20 items regarding knowledge, belief, social norms, and self-efficacy variables that corresponded to either early breastfeeding, exclusive breastfeeding, or continued breastfeeding (**Supplemental Table 1**). Knowledge was estimated by 6 items. Beliefs, social norms, and self-efficacy were measured on a 6-point Likert scale by asking mothers the extent to which they disagreed or agreed with statements. The score used was derived from the first principal component of these variables.

Variables for maternal sociodemographic characteristics included the mother’s age, education, salary, current work status, and ethnicity (majority Kinh compared with other minorities). The score used was derived from the first principal component of these variables. We also collected information relating to housing condition and assets as well as the access to improved water, sanitation, and electricity. The score used was derived from the first principal component of these variables.

#### Statistical analysis.

We used stratified analysis, multiple logistic regression, propensity score-matching analysis, and structural equation modeling. For the first 2 methods, survey commands in Stata version 13.1 (StataCorp LP) were used to account for clustering during sampling and sampling weights. The sample was weighted to 445 children/mo because children <6 mo were oversampled (crude mean child age: 8.2 mo; 56% were aged <6 mo).

First, to visualize the feeding pattern, we stratified the prevalence of infant formula and early breastfeeding cessation by infant formula feeding during the first 3 d after birth, child age (2-mo interval), and cesarean status. Nonoverlapping 95% CIs were used as a criterion for reporting statistically significant differences.

Second, we used multiple logistic regression to control for potential confounding factors to examine associations between infant formula feeding during the first 3 d after birth and the 2 outcomes: subsequent infant formula feeding and early breastfeeding cessation. We divided the scores of maternal sociodemographic characteristics and breastfeeding misconceptions into tertiles and the score of economic status into quintiles for this analysis. We also performed the analysis separately for 2 age groups (0−5 mo and 6−23 mo).

Third, to estimate the treatment effect of introducing infant formula early, we performed propensity score-matching analysis with the use of Stata version 13.1 (35). This method helped to minimize the effect of differential information bias relating to the recall of infant formula feeding during the first 3 d after birth (e.g., relating to subsequent feeding of infant formula and social desirability) and/or mothers’ commitment to breastfeeding. We created exposed and unexposed groups of newborns to infant formula during the first 3 d after birth based on the similarity of estimated propensity scores. The selection of the matched group was based on 1:1 nearest-neighbor matching within a caliper (35). The propensity scores were estimated with the use of a logit model based on various characteristics of the children (age, sex, weight-for-age *z* score), mothers (exposure to infant formula ads on television, intention to feed infant formula at birth, breastfeeding misconception scores, sociodemographic status), place and mode of delivery, household economic status, and province of residency.

Fourth, to examine potential pathways, we used Mplus 6 software (Muthén & Muthén) to perform structural equation modeling that was constructed based on a preidentified conceptual framework for the associations between outcome, exposure, and covariate variables. Standardized β coefficients are reported to facilitate comparisons between coefficients for variables that were originally on different scales. We present findings for the overall sample because stratified analysis by age group (0−5 mo and 6−23 mo) showed similar findings.

#### Ethical consideration.

The study protocol was approved by the Institutional Review Board of the Institute of Social and Medical Studies. Written informed consent was obtained from all participants.

## Results

In the weighted sample of 10,681 mothers, 93% belonged to the majority Kinh ethnicity, 20% were salaried employees, and 37% had ≥9 y of education. The mean age of the mothers was 28 y; the mean age of the children was 12.0 mo (25% were aged <6 mo). The prevalence of infant formula feeding during the first 3 d after birth was 50% and was found to be higher among women who had a cesarean delivery (78%) than those who had a vaginal delivery (43%) (95% CIs did not overlap).

### 

#### Infant formula feeding at birth and during the first 2 y.

**FIGURE 1:**
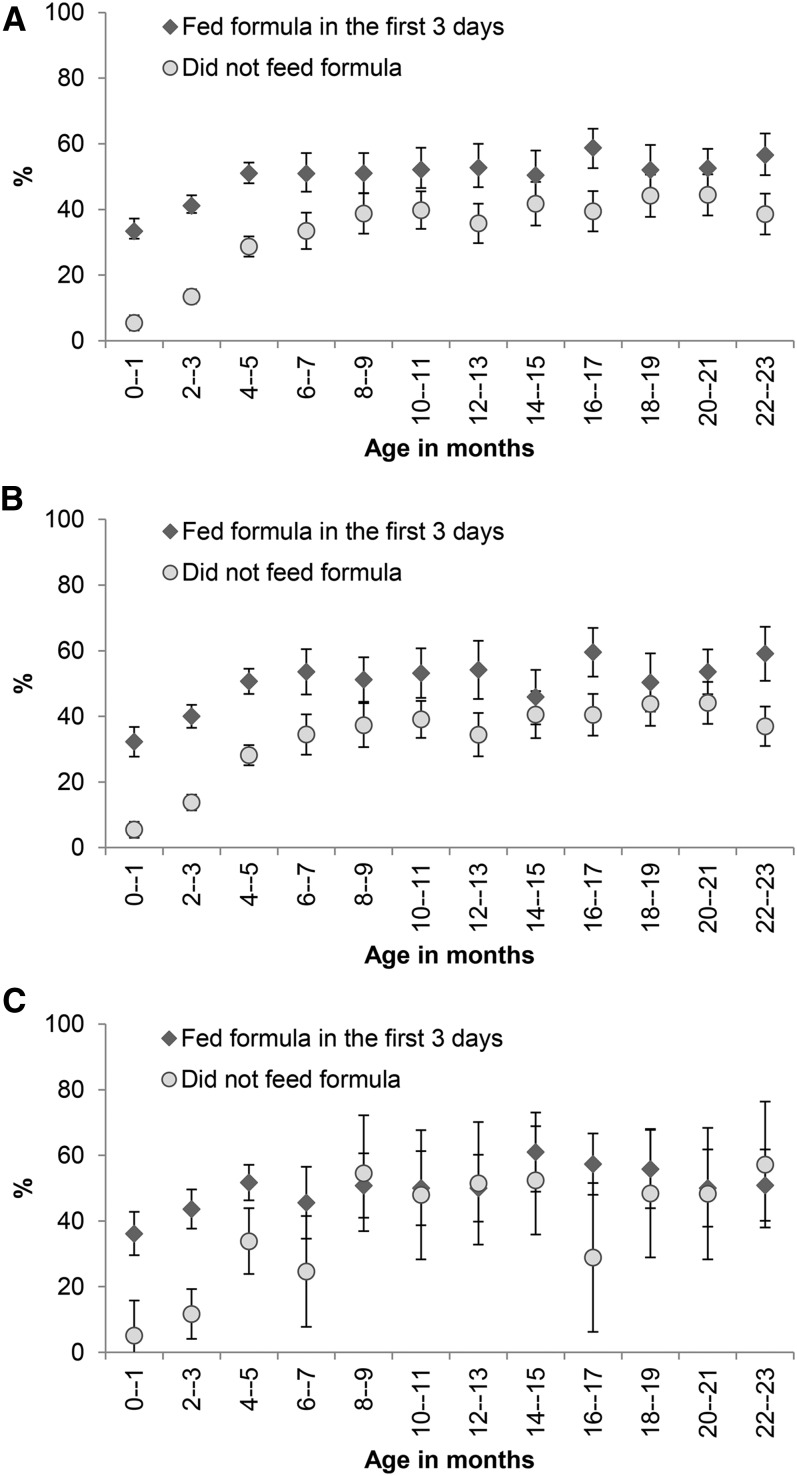
Prevalence and 95% CIs of subsequent infant formula feeding by status of infant formula feeding during the first 3 d after birth and child age overall (A), for vaginal deliveries (B), and for cesarean deliveries (C) (Alive & Thrive baseline survey). The ranges of *n* per data point were 404–486, 275–447, and 35–158, respectively.

The prevalence of subsequent infant formula feeding was 7–28% higher among children fed with infant formula during the first 3 d after birth than those who were not for those with vaginal deliveries (95% CIs did not overlap except for 3 age groups: 14−15, 18−19, and 20−21 mo) (**Figure 1**). Among mothers who reported cesarean deliveries, subsequent infant formula feeding of infants aged <6 mo was 18–32% higher among children fed with infant formula during the first 3 d after birth than those who were not (95% CIs did not overlap) (Figure 1). Propensity score-matching analysis showed that infant formula feeding in the first 3 d after birth was associated with a higher prevalence of subsequent infant formula feeding in children aged 0–23 mo (12.5%) (*P* < 0.001), 0–5 mo (13.5%) (*P* < 0.001), and 6–23 mo (3.9%) (*P* = 0.07).

In multiple logistic regression, infant formula feeding during the first 3 d after birth was associated with a higher prevalence of subsequent infant formula feeding in the overall sample (OR: 1.47; 95% CI: 1.30, 1.67), children aged 0–5 mo (OR: 2.61; 95% CI: 2.24, 3.04), and children aged 6–23 mo (OR: 1.32; 95% CI: 1.13, 1.53) (**Table 1**). Subsequent infant formula feeding was also higher among mothers who intended to feed infant formula at birth, had higher levels of misconceptions relating to exclusive and continued breastfeeding, saw infant formula promotions on television, and belonged to higher socioeconomic groups (Table 1).

**TABLE 1:** ORs of factors associated with subsequent infant formula feeding in children aged <24 mo (Alive & Thrive baseline survey)^1^

	Age, mo		
	0−23	0−5	6−23
*n*	10,681	5951	4730
Infant formula feeding during first 3 d after birth	1.47 (1.30, 1.67)***	2.61 (2.24, 3.04)***	1.32 (1.13, 1.53)***
Age, mo	1.05 (1.04, 1.05)***	1.32 (1.26, 1.38)***	1.01 (1.00, 1.03)*
Boys	1.12 (1.02, 1.23)*	1.14 (1.00, 1.30)	1.11 (0.99, 1.25)
Weight-for-age *z* score	0.91 (0.87, 0.96)***	0.84 (0.79, 0.89)***	0.92 (0.87, 0.98)**
Delivery modes			
Vaginal delivery in hospital	1.18 (1.02, 1.35)*	1.10 (0.91, 1.33)	1.20 (1.02, 1.41)*
Cesarean delivery in hospital	1.14 (0.96, 1.35)	1.21 (0.95, 1.52)	1.09 (0.90, 1.33)
Intention of feeding infant formula at birth	1.30 (1.15, 1.46)***	1.41 (1.22, 1.64)***	1.26 (1.08, 1.46)**
Misconception relating to exclusive and continued breastfeeding^2^			
Mild	1.24 (1.09, 1.41)**	1.94 (1.64, 2.29)***	1.06 (0.90, 1.25)
Severe	1.46 (1.30, 1.65)***	3.19 (2.74, 3.72)***	1.15 (0.99, 1.33)
Saw formula promotion on television within the last 30 d	1.21 (1.05, 1.39)**	0.93 (0.79, 1.09)	1.28 (1.07, 1.52)**
Maternal sociodemographic characteristics^3^			
Middle	1.07 (0.95, 1.20)	0.91 (0.76, 1.10)	1.09 (0.94, 1.25)
Higher	1.71 (1.46, 2.00)***	1.60 (1.32, 1.94)***	1.76 (1.46, 2.13)***
Household economic status^4^			
Second	1.45 (1.22, 1.73)***	1.11 (0.86, 1.43)	1.51 (1.24, 1.85)***
Third	1.71 (1.43, 2.05)***	1.43 (1.12, 1.83)**	1.77 (1.42, 2.21)***
Fourth	1.82 (1.52, 2.17)***	1.44 (1.13, 1.84)**	1.88 (1.53, 2.33)***
Fifth	2.75 (2.23, 3.40)***	2.22 (1.72, 2.86)***	2.94 (2.28, 3.78)***

1 Values are ORs (95% CIs). *,**,***Significantly different from the null value (OR = 1): *P < 0.05, **P < 0.01, and ***P < 0.001.

2 First factor from the principal component analysis of 13 items on knowledge, belief, social norms, and self-efficacy (see Supplemental Table 1).

3 First factor from the principal component analysis of mother’s age, education, salary, and Kinh ethnicity.

4 First factor from the principal component analysis of 40 items on household characteristics and assets.

#### Infant formula feeding at birth and early cessation of breastfeeding.

**FIGURE 2:**
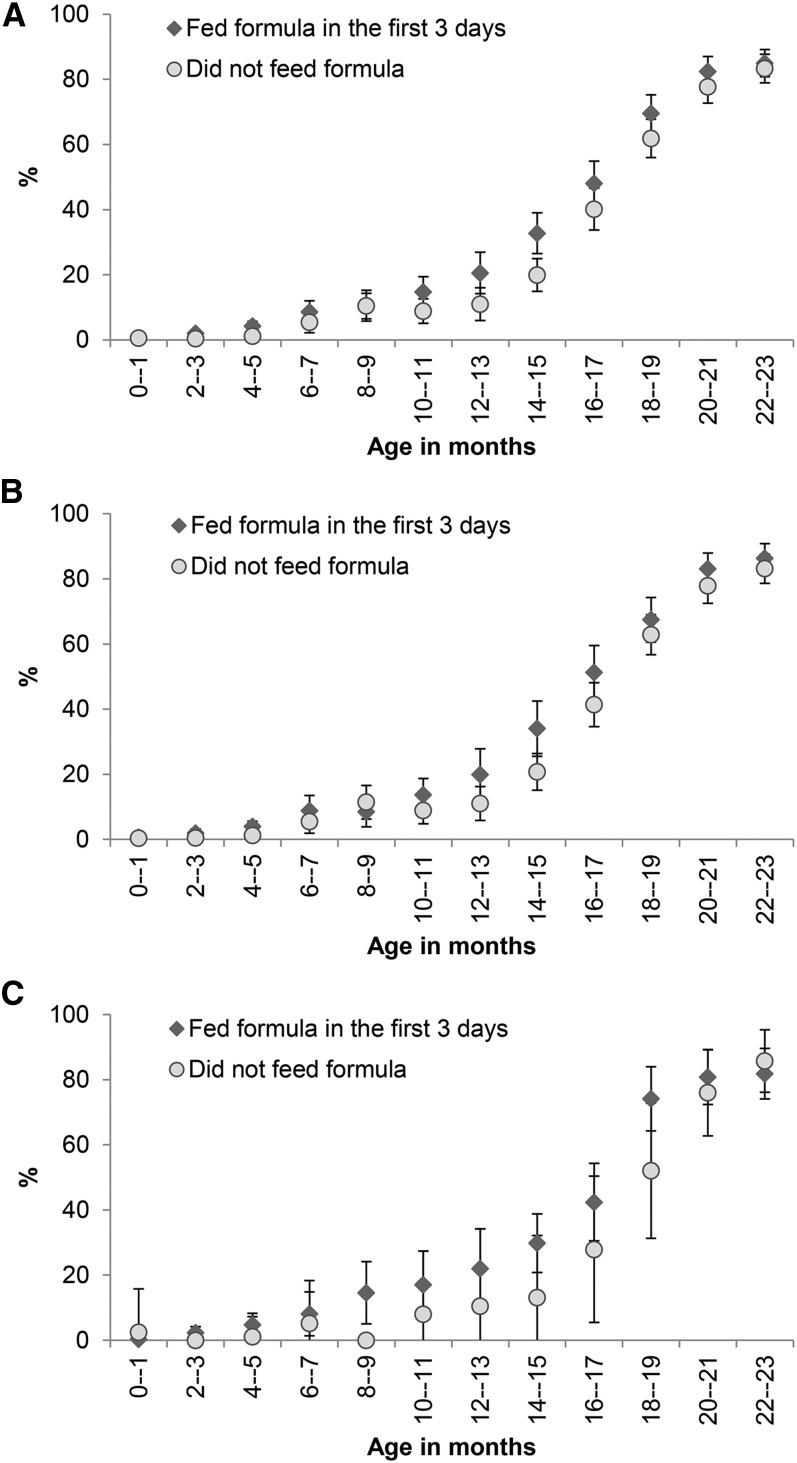
Prevalence and 95% CIs of not being breastfed on the previous day by status of infant formula feeding during the first 3 d after birth and child age overall (A), for vaginal deliveries (B), and for cesarean deliveries (C) (Alive & Thrive baseline survey). The ranges of *n* per data point were 404–486, 275–447, and 35–158, respectively.

We compared the prevalence of early breastfeeding cessation in infants fed with infant formula during the first 3 d after birth and those who were not (**Figure 2**). The propensity score-matching analysis showed that children fed with infant formula within the first 3 d had a higher tendency of early cessation of breastfeeding than those who were not in children aged 0–23 mo (2.1%) (*P* = 0.08) and 0–5 mo (0.7%) (*P =* 0.05) but not in children aged 6–23 mo (1.9%).

Infant formula feeding during the first 3 d after birth was associated with a higher prevalence of early breastfeeding cessation in the overall sample (OR: 1.33; 95% CI: 1.12, 1.59) and in children aged 0–5 mo (OR: 1.93; 95% CI: 1.03, 3.61) and 6–23 mo (OR: 1.32; 95% CI: 1.10, 1.59) (**Table 2**). In addition, early breastfeeding cessation was higher among mothers with misconceptions relating to exclusive and continued breastfeeding (Table 2).

**TABLE 2:** ORs of factors associated with not being breastfed on the previous day among children aged <24 mo (Alive & Thrive baseline survey)^1^

	Age, mo		
	0−23	0−5	6−23
*n*	10,681	5951	4730
Infant formula feeding during first 3 d after birth	1.33 (1.12, 1.59)**	1.93 (1.03, 3.61)*	1.32 (1.10, 1.59)**
Age, mo	1.39 (1.36, 1.42)***	1.51 (1.28, 1.78)***	1.40 (1.36, 1.43)***
Boys	1.11 (0.96, 1.28)	1.61 (1.03, 2.51)*	1.10 (0.94, 1.28)
Weight-for-age *z* score	1.37 (1.28, 1.48)***	1.13 (0.90, 1.43)	1.39 (1.29, 1.50)***
Delivery modes			
Vaginal delivery in hospital	0.88 (0.71, 1.09)	1.66 (0.77, 3.58)	0.87 (0.69, 1.09)
Cesarean delivery in hospital	0.79 (0.60, 1.04)	1.73 (0.84, 3.58)	0.77 (0.58, 1.03)
Intention of feeding infant formula at birth	1.05 (0.88, 1.25)	1.32 (0.86, 2.03)	1.04 (0.86, 1.25)
Misconception relating to exclusive and continued breastfeeding^2^			
Mild	1.30 (1.07, 1.57)**	1.48 (0.72, 3.05)	1.29 (1.06, 1.57)*
Severe	2.19 (1.80, 2.67)***	3.53 (2.06, 6.04)***	2.16 (1.77, 2.65)***
Saw formula promotion on television within last 30 d	0.91 (0.75, 1.12)	0.89 (0.54, 1.46)	0.91 (0.74, 1.13)
Maternal sociodemographic characteristics^3^			
Middle	0.96 (0.78, 1.17)	1.04 (0.59, 1.82)	0.96 (0.78, 1.19)
Higher	0.82 (0.65, 1.04)	1.17 (0.64, 2.13)	0.81 (0.64, 1.03)
Household economic status^4^			
Second	1.04 (0.81, 1.34)	0.80 (0.31, 2.08)	1.04 (0.81, 1.35)
Third	0.96 (0.72, 1.26)	0.94 (0.41, 2.13)	0.95 (0.72, 1.26)
Fourth	1.07 (0.81, 1.41)	1.05 (0.46, 2.41)	1.07 (0.81, 1.42)
Fifth	1.30 (0.94, 1.78)	2.00 (0.88, 4.51)	1.26 (0.91, 1.74)

1 Values are ORs (95% CIs). *,**,***Significantly different from the null value (OR = 1): *P < 0.05, **P < 0.01, and ***P < 0.001.

2 First factor from the principal component analysis of 13 items on knowledge, belief, social norms, and self-efficacy (see Supplemental Table 1).

3 First factor from the principal component analysis of mother’s age, education, salary, and Kinh ethnicity.

4 First factor from the principal component analysis of 40 items on household characteristics and assets.

#### Potential pathway from infant formula feeding at birth to subsequent feeding practices.

**FIGURE 3:**
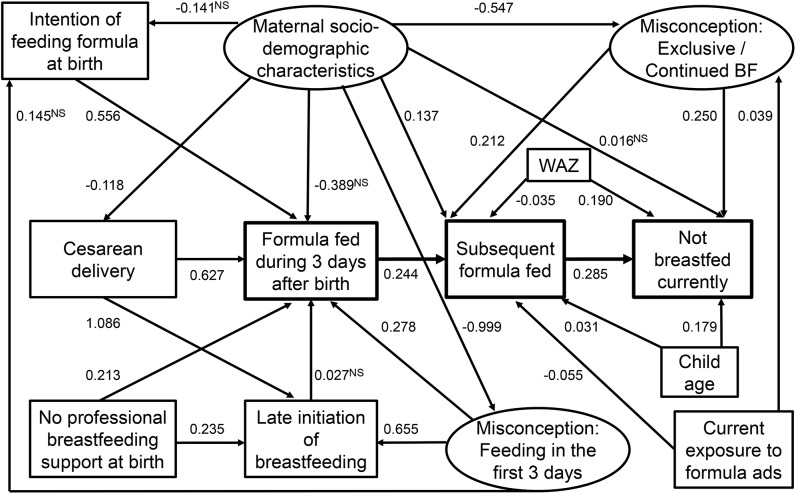
Structural equation modeling for the association among infant formula feeding during the first 3 d and subsequent infant formula feeding and early breastfeeding cessation (Alive & Thrive baseline survey). Values are standardized βs, *n* = 10,681. Ovals indicate latent variables. The latent variables were *1*) misconception relating to early and prelacteal feeding (based on 7 items), *2*) misconception relating to exclusive and continued breastfeeding (based on 13 items), and *3*) maternal sociodemographic characteristics (based on 4 items). Rectangles indicate observed variables. With the exception of 5 paths (marked NS), other paths were significant at *P* < 0.01. BF, breastfeeding; WAZ, weight-for-age z score.

Structural equation modeling showed that infant formula feeding during the first 3 d after birth was associated with higher subsequent infant formula feeding (β: 0.244; *P <* 0.001), which in turn was linked to early breastfeeding cessation (β: 0.285; *P <* 0.001) (**Figure 3**). Infant formula feeding at birth was more prevalent in mothers with misconceptions relating to feeding in the first 3 d (β: 0.278; *P <* 0.001), intention to feed infant formula at birth (β: 0.556; *P <* 0.001), cesarean delivery (β: 0.627; *P <* 0.001), and lack of professional breastfeeding support at birth (β: 0.213; *P <* 0.001). In addition, misconceptions relating to exclusive and continued breastfeeding were associated with increased subsequent infant formula feeding (β: 0.212; *P <* 0.001) and early breastfeeding cessation (β: 0.250; *P <* 0.001).

## Discussion

Infant formula feeding within the first 3 d after birth was associated with subsequent feeding practices in the context of an LMIC. Our findings support other studies that have found an association between the in-hospital introduction of infant formula and reduction of exclusive and continued breastfeeding (7, 11, 13, 16, 20–23). By matching mothers who fed infant formula to their newborns in the first 3 d with those who did not by multiple observable characteristics, we created a pseudo-RCT to yield an estimate of the treatment impact that minimized selection and information bias (35). Propensity score matching was more conservative than stratified analysis and multiple logistic regression, especially in regard to the discontinuation of breastfeeding, which confirmed that other factors, including commitment to breastfeeding and socioeconomic status (26), can play a role.

Our findings differed from the RCT in US infants born in 2 teaching hospitals in California, in which 40 exclusively breastfeeding term infants aged 1–2 d who had lost ≥5% birth weight were assigned to a control (continued breastfeeding) or intervention (giving a small amount of infant formula after each breastfeeding and discontinuing when mature milk production began) (24). Potential explanations for the difference in findings were the marked differences in the study population and setting as well as the presence of the intervention in the US study. Our findings were derived from a large-scale cross-sectional population survey in Vietnam in which we interviewed mothers with children aged 0–23 mo about their feeding practices during the first 3 d after birth and on the previous day.

There are several potential explanations for the observed association between infant formula feeding at birth and subsequent feeding practices. First, infant formula feeding in the first 3 d after birth competes with breast milk; thus, mothers produce less breast milk and then become more dependent on infant formula (6). Second, changes in sucking behavior (e.g., “nipple confusion”) because of bottle feeding with an artificial nipple can alter breast milk secretion, which leads to increased consumption of infant formula and early breastfeeding cessation (6, 36, 37). In addition, the change in infants’ taste preferences toward formula can lead to breast milk refusal and thus increased dependence on infant formula (38). Third, a preference for using infant formula has been found to be associated with both breastfeeding at birth and subsequent breastfeeding (6, 9, 11). In addition, infant formula feeding in the first 3 d after birth could alter mothers’ preferences and parenting approaches toward subsequent infant formula feeding (12, 39). Regardless of the explanation, our results re-emphasize the recommendation of minimizing the early introduction of infant formula and other prelacteal feeds for newborns (6, 8).

The decision to introduce infant formula early is typically made before labor and is influenced by factors at several levels, including the health care system, the family, and the mother (7, 40, 41). At the level of the health care system, we found that cesarean delivery and the lack of professional breastfeeding support were 2 factors associated with infant formula feeding within the first 3 d after birth. Consistent with previous findings (7, 16, 42–44), our results showed that infants born by cesarean section were considerably more likely to be fed infant formula within the first 3 d of life, which suggests the need to minimize unnecessary cesarean deliveries and to provide additional breastfeeding counseling and support to mothers who have cesarean deliveries (6, 44, 45).

Although prenatal education and support by a health professional were known to be the most effective interventions in promoting the early initiation of breastfeeding and prolonging breastfeeding duration (4, 6, 7, 9, 11, 21, 40, 46–48) and were recommended by the Vietnam Ministry of Health (49), in our sample only about half (45%) of women received professional breastfeeding advice during pregnancy and about one-third (32%) received support during the first 3 d after birth. Without proper breastfeeding information and support, mothers who experienced problems with their infant latching on or sucking, breast pain, tenderness, or cracked or painful nipples might be likely to give infant formula or other prelacteal foods to their newborns (6, 14, 48). Thus, making breastfeeding counseling and support mandatory for health workers interacting with pregnant women and new mothers is critical (6, 48, 50). Because not all health workers have sufficient knowledge and skills relating to breastfeeding advice and support, on-the-job training is recommended (4, 6, 7, 11, 21, 40, 46–48).

In relation to mothers, we found that breastfeeding misconceptions were key factors associated with infant formula feeding during the first 3 d after birth and subsequent infant formula feeding. Previous studies have indicated that mothers with breastfeeding perceptions and social norms that go against the recommended practices were more likely to feed their children infant formula while in a maternity hospital or at discharge (8, 9, 51) and then discontinue breastfeeding early (20, 51). Similar to earlier studies (11, 16, 22, 47), we found that the intention to feed infant formula to the newborn at birth was considerably related to infant formula feeding during the first 3 d after birth. Even when a mother decided to breastfeed her newborn exclusively and received good breastfeeding support from health workers, she may have been affected by advice from people she respects and believes such as other mothers, other women, senior or experienced friends, her husband, etc. (29, 41, 52–54). Because these other sources might not have accurate information or beliefs that align with recommended practices of early, exclusive, and prolonged breastfeeding and thus support infant formula (22, 52–54), interventions to change social norms are needed. In addition to changing social norms, interventions should address competing messages from formula companies that are regularly promoted through advertisements, editorial content, community and school events, and sponsorship to health workers and facilities (52–54).

Mothers’ recall of infant formula feeding within the first 3 d after birth might be a source of error, especially when the child was aged >6 mo (25). Because women in Vietnam generally only have 1–2 children, we believe that the experiences during childbirth (place of delivery, mode of delivery, breastfeeding initiation, feeding of infant formula, receiving breastfeeding support) are more easily remembered in Vietnam than in a setting with higher fertility. In addition, mothers in Vietnam or other LMICs might remember infant formula feeding during the first 3 d better than those from more developed countries because they need to buy a high-cost formula for their newborns from their pocket money and recall this practice with other special events such as cesarean delivery or difficulty in breastfeeding their newborns. The subsequent feeding practice (e.g., feeding of formula) and/or social desirability might affect recall (25). With the propensity scoring, we matched exposed and unexposed groups by multiple known characteristics and thus minimized the potential effects of information bias resulting from systematic differences between the exposed and comparison groups.

In the context of an LMIC, feeding infant formula to newborns within the first 3 d after birth was associated with increased subsequent formula feeding and early cessation of breastfeeding, which underscores the need to make early, exclusive breastfeeding normative and to create environments that support it. These findings from a large-population survey with a high response rate can be generalized to mothers in Vietnam and are applicable to mothers who live in similar settings in which there are insufficient resources for promoting breastfeeding and in which baby food companies are substantially influential.
